# Supplementary material for: Differential Leukocyte and Platelet Profiles in Distinct Models of Traumatic Brain Injury
Source: Cells. 2021 Feb 26;10(3):500. doi: 10.3390/cells10030500 (PMC7996744; doi:10.3390/cells10030500)
Supplement: Supplementary file 1 [file cells-10-00500-s001.pdf]

Differential Leukocyte and Platelet Profiles in Distinct Models of Traumatic Brain Injury

Brad Hubbard [1,2,3,4], Meenakshi Banerjee [5,†], Hemendra Vekaria [1], Kanakanagavalli Shravani Prakhya [5], Smita Joshi [4,5], Qing Jun Wang [6], Kathryn E. Saatman [1,2], Sidney W. Whiteheart [4,5], Patrick G. Sullivan [1,3,4,*]

Supplementary Materials:

This replication study was performed using the Seahorse XFe24, therefore utilizing more platelets than with the Seahorse XFe96. This is an independent replication demonstrating increased RCR at 24h post-CCI in mice.

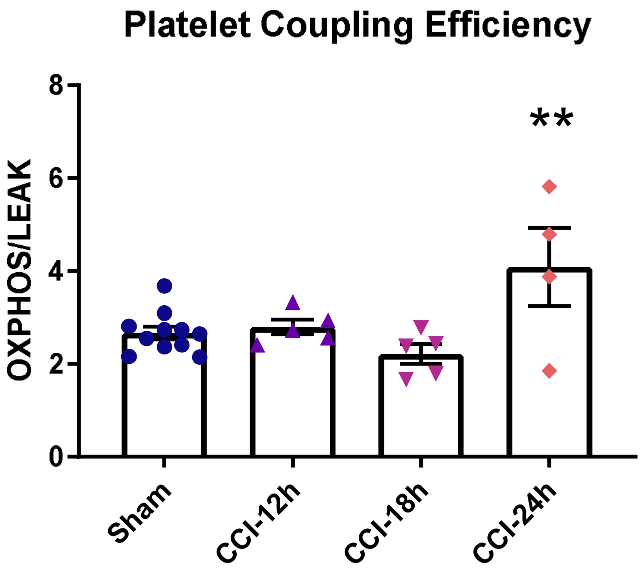

Figure S1. Dynamic changes in platelet coupling efficiency acutely following contusion brain injury. Mice received either sham injury or severe CCI followed by euthanasia at either 12, 18 or 24 h post-injury. Isolated platelets underwent respirometry using the Seahorse XFe24. Platelet coupling efficiency was increased 24 h after CCI compared to sham. t= 2.656; * p= 0.012. There was a significant increase in platelet coupling efficiency 24 h after CCI compared to sham. $F_{(4, 25)}$ = 4.349; ** p= 0.002 compared to sham. n = 4–11/group, Mean ± SEM.

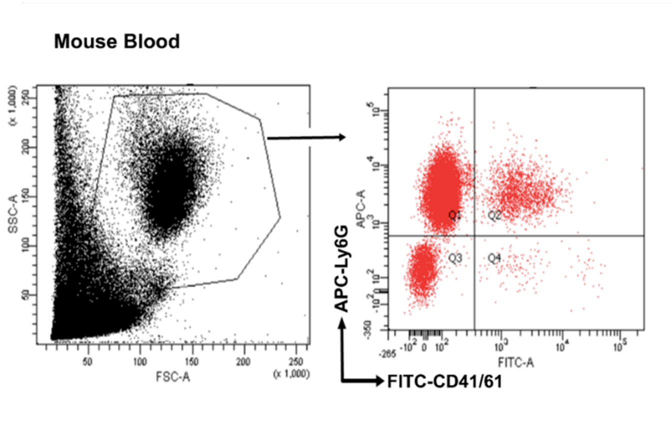

Figure S2. Schematic for platelet-neutrophil aggregates in whole mouse blood. Whole mouse blood was incubated with FITC-anti-CD41/61 (for platelets) and APC-anti- Ly6G (for neutrophils) antibodies for 30 min at RT. Using BD LSRII™ flow cytometer, double-positive platelet-neutrophil

aggregates were acquired by gating around 50,000 granulocytes (marked by polyhedral) and dot-plots were created from that (denoted by arrow). Quadrants were drawn to show single-positive and double-positive CD41/61^{+}-Ly6G^{+} events. Data were then analyzed using the FlowJo software (v7.6.5) and plotted as percentages of events in each quadrant over total granulocytes.
